# Supplementary material for: The Impact of a Web-Based Lifestyle Educational Program (‘Living Better’) Reintervention on Hypertensive Overweight or Obese Patients
Source: Nutrients. 2022 May 27;14(11):2235. doi: 10.3390/nu14112235 (PMC9182666; doi:10.3390/nu14112235)
Supplement: Supplementary file 1 [file nutrients-14-02235-s001.zip › nutrients-1731980-supplementary.pdf]

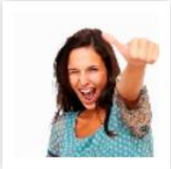

M0-Welcome

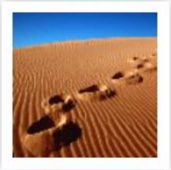

M1-Motivation for change

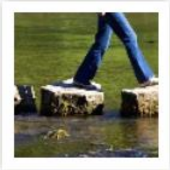

M2-Education in nutrition and physical activity

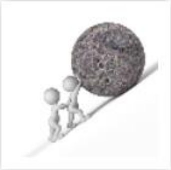

M3-Strategies to overcome possible barriers

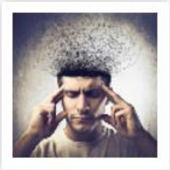

M4-Establishing diet and physical activity objectives and goals, and modification of irrational thoughts

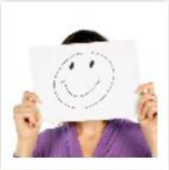

M5-Establishing diet and physical activity goals and objectives, and strategies to overcome obstacles (emotional regulation, emotional eating, and self-control)

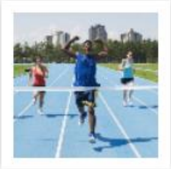

M6-Strategies to overcome obstacles and solve problems

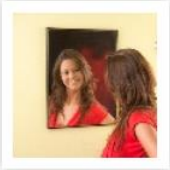

M7-Intervention for difficulties with body image and assertiveness

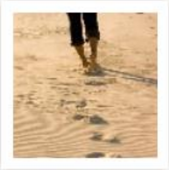

M8-Prevention of relapses

Figure S1. 'Living Better' program modules.
